# Temporomandibular joint degeneration arises spontaneously in STR/ort mice and is prevented by targeted aggrecanase inhibition

**DOI:** 10.1016/j.ocarto.2025.100599

**Published:** 2025-03-11

**Authors:** Kazuhiro Ooi, Kazuhiro Yamamoto, Yutaka Kobayashi, Behzad Javaheri, Anders Jensen, Ioannis Kanakis, Takao Sakai, Fadi Jarad, Hiroyuki Nakamura, Andrew A. Pitsillides, Shuichi Kawashiri, George Bou-Gharios

**Affiliations:** a Department of Oral and Maxillofacial Surgery, Graduate School of Medical Science, Kanazawa University, Ishikawa, Japan; b Department of Musculoskeletal and Aging Science, Institute of Life Course and Medical Sciences, University of Liverpool, Liverpool, United Kingdom; c Skeletal Biology Group, Department of Comparative Biomedical Sciences, Royal Veterinary College, London, United Kingdom; d Chester Medical School, Faculty of Medicine and Life Sciences, University of Chester, Chester, United Kingdom; e Department of Diagnostic Pathology, Faculty of Medicine, Fujita Health University, Aichi, Japan; f Department of School of Dentistry, Faculty of Health and Life Sciences, University of Liverpool, Liverpool, United Kingdom; g Department of Oral and Maxillofacial Surgery, Graduate School of Medical Science, Ryukyu University, Okinawa, Japan

**Keywords:** TIMP-3, [-1A] TIMP-3, Aggrecanases, MMPs, Temporomandibular joint osteoarthritis, Cartilage degradation

## Abstract

**Objective:**

Temporomandibular joint osteoarthritis (TMJ-OA) is painful and causes masticatory dysfunction, but current treatment is limited to symptom relief due to an incomplete appreciation of aetiology. Herein, we develop morphological and histological methods for quantitative evaluation of TMJ-OA severity and examine whether STR/Ort mice, which are genetically predisposed to spontaneous knee OA, exhibit protection against TMJ-OA upon genetic gain-of-function modification of an aggrecanase-selective mutant of tissue inhibitor of metalloproteinase (TIMP)-3.

**Design:**

We established morphological changes in mandibular condylar head adapted from human TMJ-OA criteria, and developed and verified the utility of TMJ-OA histological damage scoring adapted from the OARSI system. Mutant TIMP3 containing an extra alanine at the N-Terminus ([-1A] TIMP-3 was overexpressed in STR/Ort and CBA mice. Morphological changes in mandibular condyle and TMJ cartilage degradation were evaluated and quantified using micro-CT and histology in mice aged 10, 20 and 40 weeks.

**Results:**

Whilst no evidence of TMJ-OA was observed in STR/Ort mice aged 10 weeks, bone erosion and osteophyte formation appeared in the mandibular condyle by 20 weeks, with remarkable deformity and bone resorption at 40 weeks in STR/Ort, but not the parental CBA strain. TMJ-OA was less severe in 40 week-old [-1A]TIMP-3 overexpressing STR/Ort and CBA compared to wild-type mice.

**Conclusions:**

Using our new mouse TMJ-OA scoring system we have found that OA affects joints other than the knee in the STR/Ort strain. Genetic gain-of-function modification of STR/Ort mice with an aggrecanase-selective mutant of tissue inhibitor of metalloproteinase (TIMP)-3 also affords in vivo chondroprotection against this TMJ-OA.

## 1 Introduction

The temporomandibular joint (TMJ) is a diarthrodial mandibular articulation at the cranial base essential for functions such as mastication and speech. The human TMJ is commonly affected by osteoarthritis (OA), with resultant pain and severely limited mouth opening in 8–16 ​% of the human population [1,2]. Temporomandibular joint osteoarthritis (TMJ-OA) is characterised by articular degeneration, destruction of the articular disc and by mandibular condylar deformity that can, in severe cases, result in anterior open bite [3]. Clinical TMJ-OA diagnosis primarily relies on radiographic features of the condyle and articular eminence, including erosive bone resorption, sclerosis, atrophy, osteophyte formation, and cyst-like changes [4]. Whilst these morphological features can help in the assessment of human TMJ-OA severity, the aetiology of these pathological changes remains undefined. Progress in defining the mechanisms underpinning TMJ-OA have been limited, at least partly, by a paucity of spontaneous animal models in which joint pathological processes and molecular aetiology have verified resemblance to human disease. We therefore explore, herein, whether TMJ-OA develops in a mouse strain with known genetic predisposition to spontaneous, progressive knee joint OA [5].

Current TMJ-OA treatment options include physical therapy, occlusal splints, non-steroidal anti-inflammatory drugs and arthrocentesis with intra-articular corticosteroid or lubrication therapy [6]. Surgical joint replacement with autologous bone or an artificial joint is the final option, but in severe cases, it may not alleviate functional impairment or the intractable pain [6]. These options are mostly palliative because TMJ-OA aetiology is likely complex and multifactorial; and currently undefined. TMJ overloading due to occlusal disorders or chewing habits, such as bruxism and clenching, is known to cause structural changes leading to TMJ-OA [7]. As the underlying aetiopathological mechanisms remain incompletely understood, their elucidation is crucial for developing definitive treatment or prevention strategies.

The articular surface of the mandibular condyle is covered by cartilage that is composed mainly of collagen fibers and proteoglycans, however, this TMJ cartilage differs from articular cartilage in other joints due to the predominant presence of type I collagen; which is a significant component of the superficial zone of articular cartilage, though type II collagen (the dominant type in hyaline cartilage) is dominant in the mature and hypertrophic zones [8]. This construction results in a viscoelastic response to loading and enables the cartilage to play an important role as a stress absorber during function [9]. Other differences between the mandibular condylar cartilage and articular cartilage include rapid chondrocyte hypertrophy with the overlapping expression of collagen type I and the absence of growth factors GDF-5 and GDF-69; specific osteopontin and bone sialoprotein expression. In addition, the bone of the mandibular condyles beneath the fibrocartilage, is particularly susceptible to inflammatory damage [4]. It is unclear, however, whether these distinctions in mandibular condyle articular composition require that specific therapeutic approaches be developed to prevent TMJ-OA.

Little is known about the mechanism underlying the destruction of mandibular condylar cartilage in TMJ-OA. In recent years, therapies such as intraarticular platelet-rich plasma, hyaluronic acid, and mesenchymal stem cell-based treatment have shown promising results with respect to structural regeneration or protection against further damage in TMJ OA [10]. Synovial fluid from TMJ-OA patients contains elevated levels of interleukins, matrix metalloproteinases (MMPs, e.g. MMP-1, MMP-8 and MMP-13 collagenases) and aggrecanases (a disintegrin and metalloproteinase with thrombospondin motifs (ADAMTS)-4 and ADAMTS-5). All of these proteinases can contribute to joint destruction [[11], [12], [13]] and it is now recognized that collagenases and aggrecanases are highly expressed in the articular disc and synovial tissues of TMJ-OA patients [[14], [15], [16]]. Furthermore, our recent study confirmed aggrecanases were expressed in the chondrocytes in the superficial zones of the mandibular cartilage in animal model [17]. However, that there is no confirmation that these aggrecanases are primarily involved in TMJ tissue degradation, and this question is particularly pertinent since TMJ articular composition is not identical to diarthrodial joints of the appendicular skeleton.

Mouse TMJ-OA models can be established by a range of methods, each with expected advantages and disadvantages. For example, TMJ-OA can be induced surgically, though intra-articular injection [[18], [19], [20]] and by inducing ‘crossbite’, yet natural TMJ-OA models are atypical and require quantitative evaluation in order to confirm their validity in tracking spontaneous OA progression via the use of morphological and histological analyses [21]. The major advantage of models in which OA is induced is that timing of this induction can be controlled. However, the major advantage of using animals in which OA arises spontaneously is that they do not involve interventions that can complicate interpretation. Natural TMJ-OA models are also thought to be useful for studying the differences in onset factors between knee or hip joints and TMJ. The STR/Ort mouse strain is a well-established genetic model of spontaneous knee joint OA [22] in which reports of TMJ-OA remain rare [23]. Given the distinct structural composition of TMJ cartilage, we explored whether aggrecanase inhibition would protect the mandibular condylar cartilage against OA-related progressive deterioration as has already been shown in articular cartilage of the knee [24,25]. We therefore explore, herein, whether OA arises spontaneously in the STR/Ort mouse TMJ and also whether selective aggrecanase inhibition, via targeted [-1A] TIMP-3 [26] overexpression, extends protection to such TMJ joint degeneration. Our studies define a new mouse model in which genetic susceptibility predisposes to TMJ-OA in the absence of any traumatic loading in the STR/Ort strain and reaffirms, through selective aggrecanase inhibition, the future utility of this mouse strain in tracking the efficacy of any new therapeutic candidates for TMJ-OA.

## 2 Materials and methods

### 2.1 Generation of lentiviral vectors to overexpress [-1A] TIMP-3

We generated lentiviral vectors to overexpress [-1A] TIMP-3 using methods described by Kanakis et al. [26]. The lentivirus vector was constructed by cloning [-1A] TIMP-3 with a FLAG tag into the *Eco*RV‒*Sal*I site of the pCCL-expressing vector driven by the elongation factor 1α (EF-1α) promoter. The construct also contained nerve growth factor receptor as a marker. To produce the virus, 293T17 ​cells were co-transfected with transfer and packaging vectors by electroporation using a Neon transfection system (Invitrogen). Viral supernatants were harvested 48 and 72 ​h after transfection and concentrated by ultracentrifugation at 10,000 ​g for 2 ​h. The viral pellet was collected and dissolved in phosphate buffered saline. Transient transduction was performed at a multiplicity of infection of 10 in HEK293 and HTB94 ​cell lines to test integration efficacy and overexpression capacity. Transfection efficiency was estimated using flow cytometry with allophycocyanin-conjugated anti-NGFR. Protein levels of [-1A] TIMP-3 were assessed by Western Blotting with either anti–TIMP-3 antibody or rabbit anti–β-actin (ab8227; Abcam).

### 2.2 Mice and generation of [-1A] TIMP-3 transgenic mice

[-1A] TIMP-3 overexpressing STR/Ort and CBA mouse strains were generated as previously described [27]. A STR/Ort mouse colony maintained at Royal Veterinary College London was used to generate transgenic mice. Each fertilized embryo was infected using 100 lentiviral particles (EF- 1α[-1A] TIMP-3) per embryo. Newborn transgenic mice were genotyped by quantitative polymerase chain reaction using the reporter gene LacZ and the [-1A] TIMP-3 insert (Supplementary information; http://onlinelibrary.wiley.com/doi/10.1002/art.40765/abstract). All experimental protocols complied with the UK Animals (Scientific Procedures) Act 1986 regulations and were approved by the ethics committee of the Kanazawa University Graduate School of Medical Science. We analyzed TMJ from STR/Ort mice and CBA mice at 10, 20 and 40 weeks of age. These ages were chosen because they represent ages at which OA in the knee show this progression: 10 (pre-OA), 20 (OA onset) and 40 weeks (advanced OA) of age. CBA mice were studied at the same ages to allow for direct comparison [28]. To explore whether protection against TMJ-OA by [-1A] TIMP-3 was restricted to the genetically-susceptible OA-prone STR/Ort, we also investigated the effects of transgene overexpression in CBA mice. Age-matched untreated STR/Ort mice and untreated CBA mice served as controls; CBA mice are a parental background strain of STR/Ort mice.

### 2.3 Micro‒computed tomography analysis and evaluation of morphological changes in mandibular condylar head using a newly developed scoring system

**Fig. 1:**
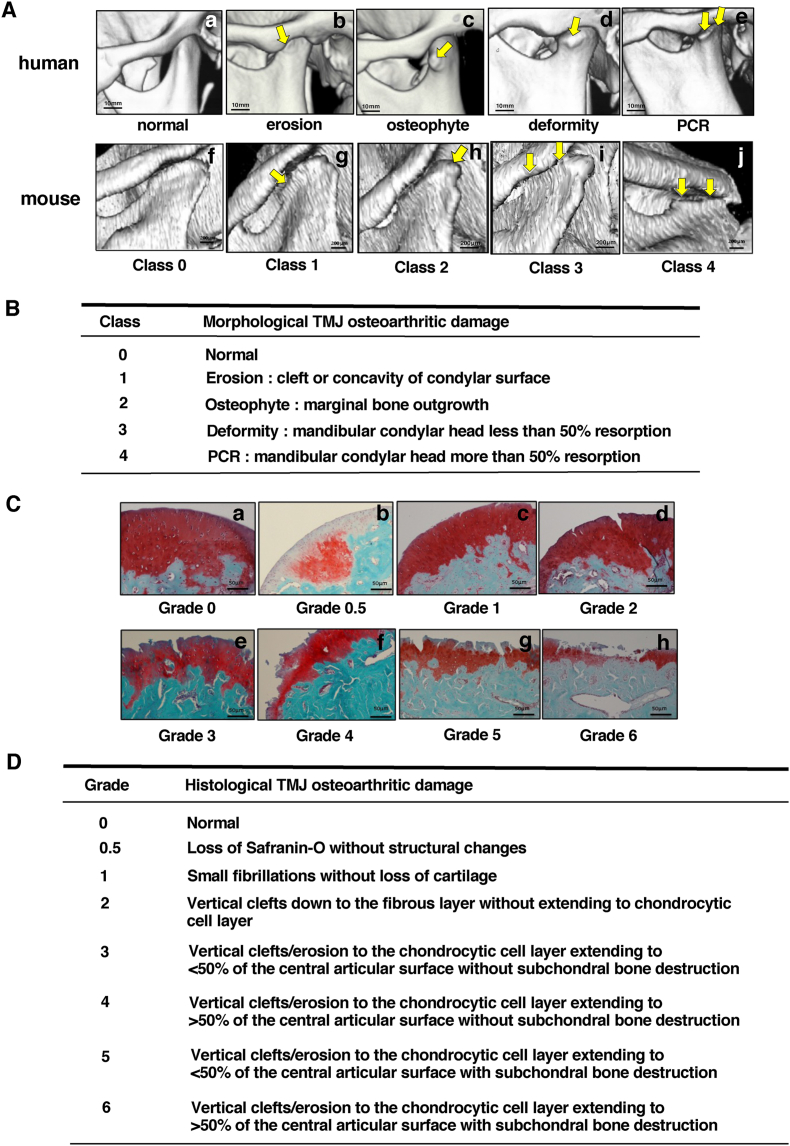
**Typical human morphological TMJ-OA classification and morphological and histological mouse TMJ scoring systems.** (A) μCT of human TMJ condylar structural changes: healthy TMJ (normal, a); mandibular condylar surface is eroded (erosion, b); formation of osteophyte observed on the lateral edge of mandibular condyle (osteophyte, c), shortening of the mandibular condyle (deformity, d); progressive condylar resorption (PCR, e). Typical 3D μCT images of OA classification for mouse TMJ: (Class 0 (f), 1(g), 2(h), 3(i), 4(j)). (B) Morphological TMJ osteoarthritic damage scoring system for mouse (Class 0; normal, 1; erosion, 2; osteophyte, 3; deformity, 4; PCR). (C) Typical histological mandibular condylar surface images (Safranin-O/Fast green staining) of TMJ-OA in mouse (Grade 0(a), 0.5(b), 1(c),2(d),3(e),4(f),5(g),6(h)). (D) Histological TMJ osteoarthritic damage grading system in mouse (Grade 0; normal to Grade 6 depends on severity of loss of cartilage and vertical clefts/erosion to the chondrocytic cell layer extending to central articular with or without subchondral bone destruction).

The mouse head was dissected, and the mandibles were divided into right and left sides for imaging with Skyscan 1272 micro‒computed tomography (μCT) scanner (Bruker). The imaging parameters were set at 50 ​kV, 0.5 aluminium filter, 200 ​mA, voxel size 5.00 ​μm, and 0.3° rotation angle. Data sets were reconstructed using NRecon software, and 3-dimensional volumes of interest were selected by DataViewer and CTAn software. μCT analysis was performed on the TMJ of otherwise untreated (non-transgenic) ‘wild type’ STR/Ort mice, WT CBA mice and [-1A] TIMP-3 overexpressing transgenic STR/Ort mice at 10, 20 and 40 weeks of age. Morphological changes indicative of TMJ-OA were scored using a five-level system (Fig. 1A/B), adapted from human TMJ-OA criteria. Two experienced researchers conducted the scoring in a blinded manner and the scores were calculated as the average of the scores recorded across the five levels.

### 2.4 Histological damage analysis and the evaluation of mandibular condylar head using a newly developed scoring system

After μCT scanning, formalin-fixed TMJ samples were decalcified with mixed solution of formic acid and EDTA (Formical-2000™) for 24 ​h and then stored in 70 ​% ethanol until processing. Samples were embedded in paraffin wax and sectioned in the sagittal plane at 5 ​μm thickness through the entire joint. Proteoglycan loss and cartilage degradation of the mandibular condylar head was assessed using safranin-O fast green staining of sections collected at 25 ​μm intervals. Histological TMJ-OA severity was scored on an eight-level scale (Fig. 1C/D), based on a modified version of Osteoarthritis Research Society International (OARSI) recommendations [29]. Two experienced researchers performed the scoring in a blinded manner and the final scores were calculated as the average of the scores recorded at eight levels.

### 2.5 Statistical analysis

Age-related changes and the effect of overexpressing [-1A] TIMP-3 were statistically analyzed in each mouse model. Gender and unilateral differences were also statistically analyzed in STR/ort mouse. All data were analyzed with GraphPad Prism 9 software and expressed as the mean ​± ​SD. Morphological and histological OA severity scores between STR/Ort, CBA and [-1A] TIMP-3 overexpressing transgenic STR/Ort mice at each age were analyzed using Mann-Whitney *U* test or. ANOVA test. In all cases, *P* value less than 0.05 considered significant.

## 3 Results

Application of a new morphological and histological scoring system allows quantitative evaluation of TMJ-OA and reveals an age-associated progression in STR/Ort mice.

An established classification system for human TMJ-OA severity based upon evaluation of μCT scan images in which OA is graded by morphological criteria on a 5-point scale has been developed; from normal to erosion, osteophyte formation, deformity, and progressive condylar resorption based on CT surface imaging [16]. We therefore used a similar 3D μCT classification system with aligned morphological changes (Fig. 1A) in images from a large number of STR/Ort mouse TMJ (n ​= ​60) to find that mandibular condylar bone changes could be classified on a 5-point scale: Class 0: normal, Class 1: cleft or concavity of condylar surface, Class 2: marginal bone growth, Class 3: resorption of <50 % of the condylar head, Class 4: resorption of >50 ​% of the condylar head (Fig. 1B).

These morphological changes in TMJ-OA prompted us to apply histological methods for detailed quantitative analysis in STR/Ort mice. Using a modified OARSI scoring system, we graded severity based on articular cartilage surface and internal structural integrity, including subchondral bone changes on an 8-point scale, from normal to progressive loss of Safranin-O without structural changes, small fibrillations without cartilage loss, vertical clefts down to the fibrous layer without extending to the chondrocytic cell layer, 50 ​% less or over vertical clefts/erosion to the chondrocytic cell layer extending to the central articular surface, with or without subchondral destruction at the mandibular condyle TMJ surface in sections stained with Safranin-O/Fast green (Fig. 1C and D). This showed that combined morphological/histological scoring provides a robust framework for assessing mouse TMJ-OA severity, facilitating precise and detailed appraisal of disease progression.

**Fig. 2:**
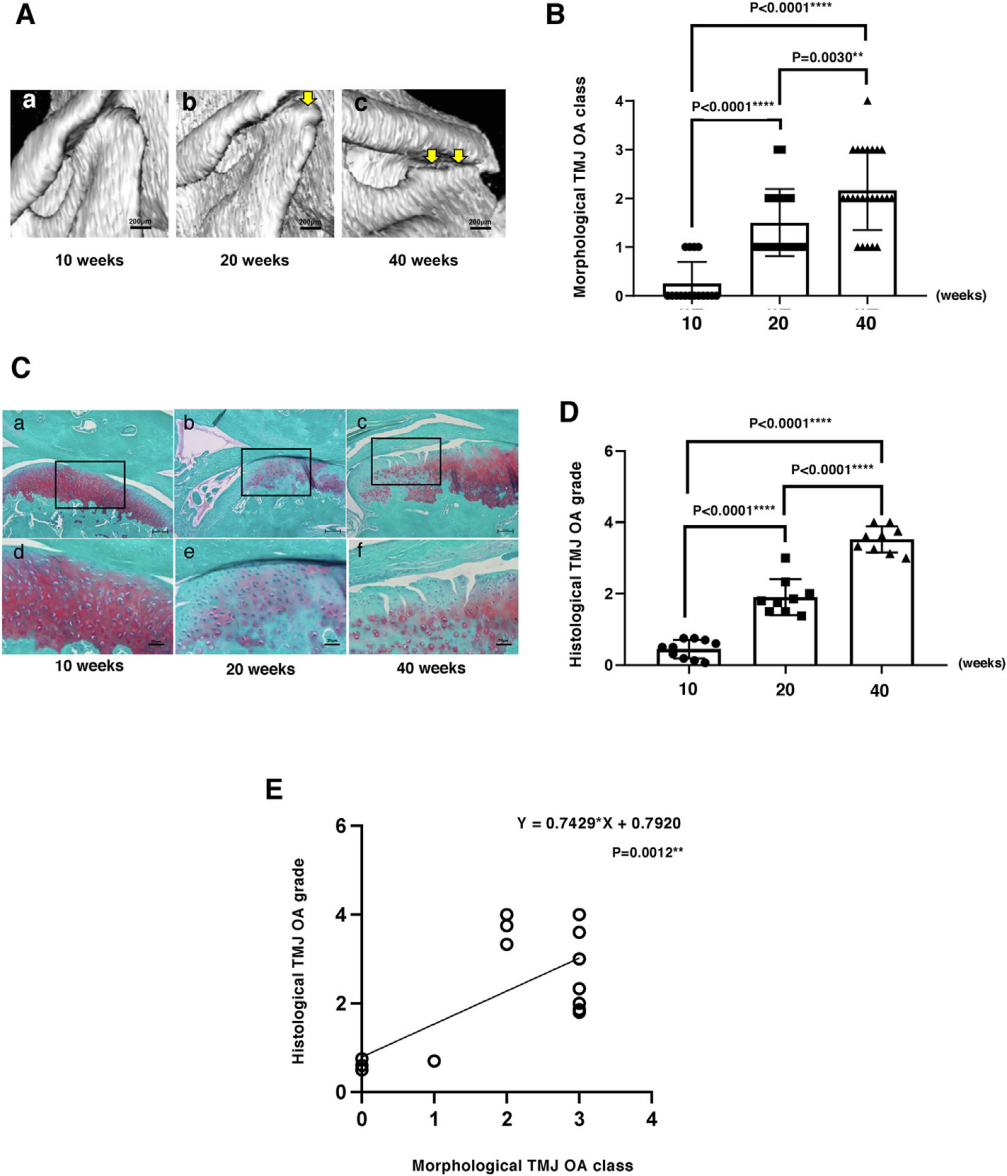
**STR/Ort mice develop age-dependent TMJ-OA.** (A) While class 0 ​at 10 weeks show the majority of condyles appear normal (a), 20 weeks show osteophytes (b) Most severe damage 3D CT images of mandibular condylar head is seen by 40 weeks it reaches progressive condylar resorption (c). (B) Morphological score of mandibular condylar head were statistically significantly increased according to aging (C) Typical histological images of mandibular condylar head (a–c: bar is 200 ​μm, d–f: bar 50 ​μm). Histological images of the same condyles appearing in μCT showed grade 0 was observed in of mandibular condylar head at 10 weeks (a,d). Grade 2 was observed in histological images of mandibular condylar head at 20 weeks (b,e). Grade 6 was observed in histological images of mandibular condylar head at 40 weeks (c,f). (D) Histological score of mandibular condylar head were statistically significantly increased according to aging. (E) Histological TMJ-OA was statistical significantly increase according to morphological TMJ-OA class increasing.

Using CT-based scoring we observed that Class 0 TMJ morphology, indicating normal joints, was predominant in 10-week-old STR/Ort mice(n ​= ​10). By 20 weeks(n ​= ​9), most mice exhibited Class 2 morphology with osteophytic changes, and by 40 weeks(n ​= ​9), a very severe joint deterioration; Class 4 with evident progressive condylar resorption was also observed (Fig. 2A). These data show a statistically significant increase in morphological TMJ scores with age in STR/Ort mice (P ​< ​0.0001; Fig. 2B), mirroring human TMJ-OA progression. Histological grading of the same joints confirmed that Grade 0, 2 and 4 predominated in 10, 20 and 40-week-old STR/Ort mice, respectively (Fig. 2C and D) demonstrating significant increase in TMJ-OA severity with ageing (P ​< ​0.0001; Fig. 2D) and strong correlation with CT-based morphological classification (P ​= ​0.0012; Fig. 2E). Together this further validates the robustness of this combined scoring and confirms that STR/Ort mice spontaneously acquire a self-progressing OA in the TMJ.

### 3.1 STR/Ort mice exhibit sex and asymmetrical morphological and histopathological differences in TMJ-OA

**Fig. 3:**
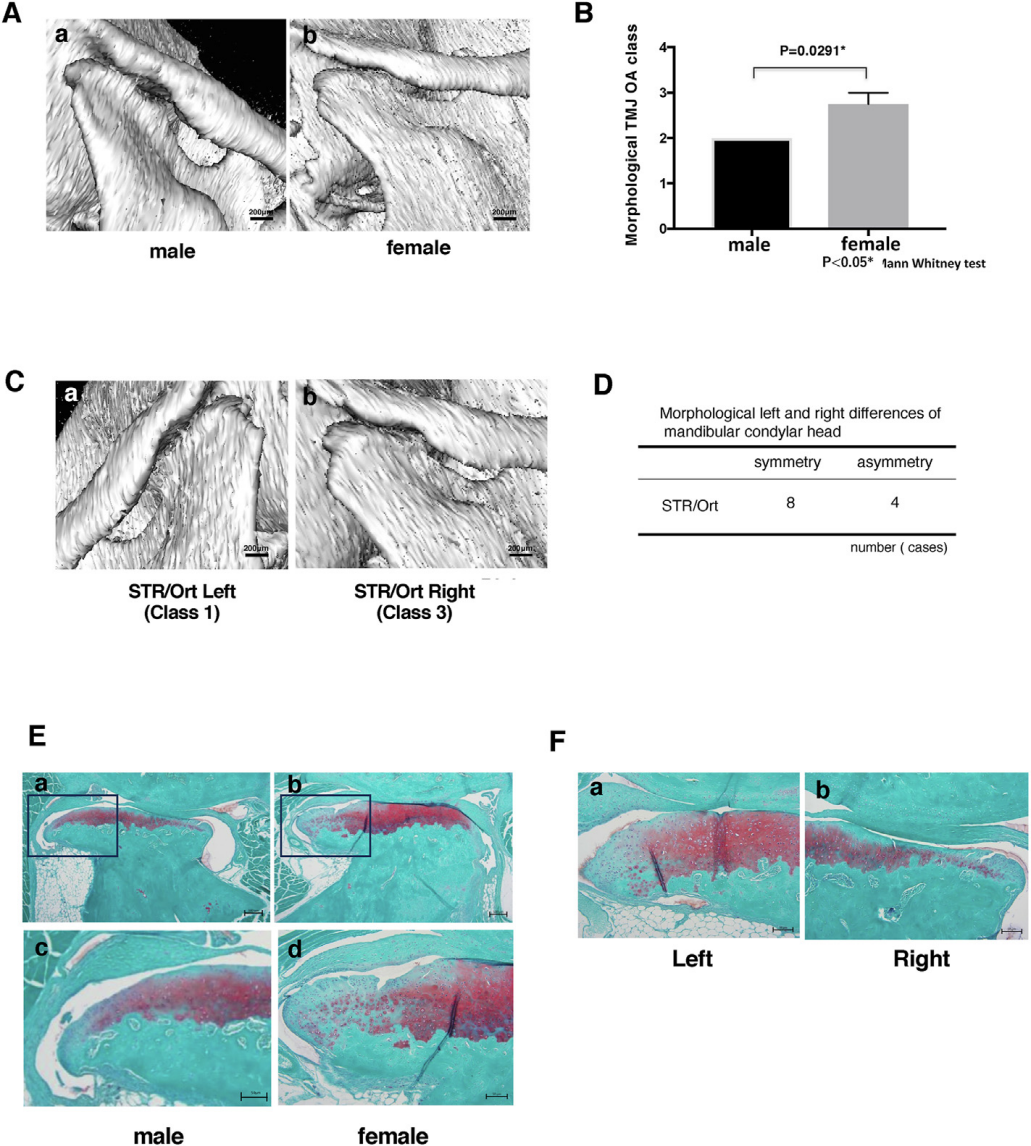
**Sex and asymmetrical morphological differences of TMJ-OA in STR/Ort mice.** (A) Typical 3D CT images of mandibular condylar head of untreated STR/ort male and female. (B) Female mice TMJ score was higher than male of TMJ OA score in STR/Ort mice at 40weeks (C) it was noted that asymmetry exist between left and right TMJ in the same animal. Mandibular condylar head showing class 1 on the left side compared to class 3 on the right (D) Mandibular condylar asymmetry was observed in 4 (40 weeks old STR/ort) out of 12 mice examined. Similar features were also observed in sagittal histological sections stained with Safronin O and fast green (E, F). In E, higher magnification of the area depicted in the black rectangles are highlighted in cand d.

We also investigated sex differences in TMJ-OA incidence in STR/Ort mice, which is typically higher in males for knee OA [25]. Interestingly, at 40 weeks, female mice exhibit higher CT-based TMJ-OA classification than males, indicating reverse sexual dimorphism in TMJ-OA susceptibility in this strain (n ​= ​0.0291; Fig. 3 A/B). Examination of left and right TMJs revealed some asymmetry: 4 out of 12 left/right TMJ pairs in 40-week-old STR/Ort mice showed morphological differences greater than one class (Fig. 3C/D). Similar features were also observed in sagittal histological sections stained with Safronin O and fast green (Fig. 3 E, F).

### 3.2 [-1A] TIMP-3 prevents the development of TMJ-OA in STR/ort mice at 40 weeks

**Fig. 4:**
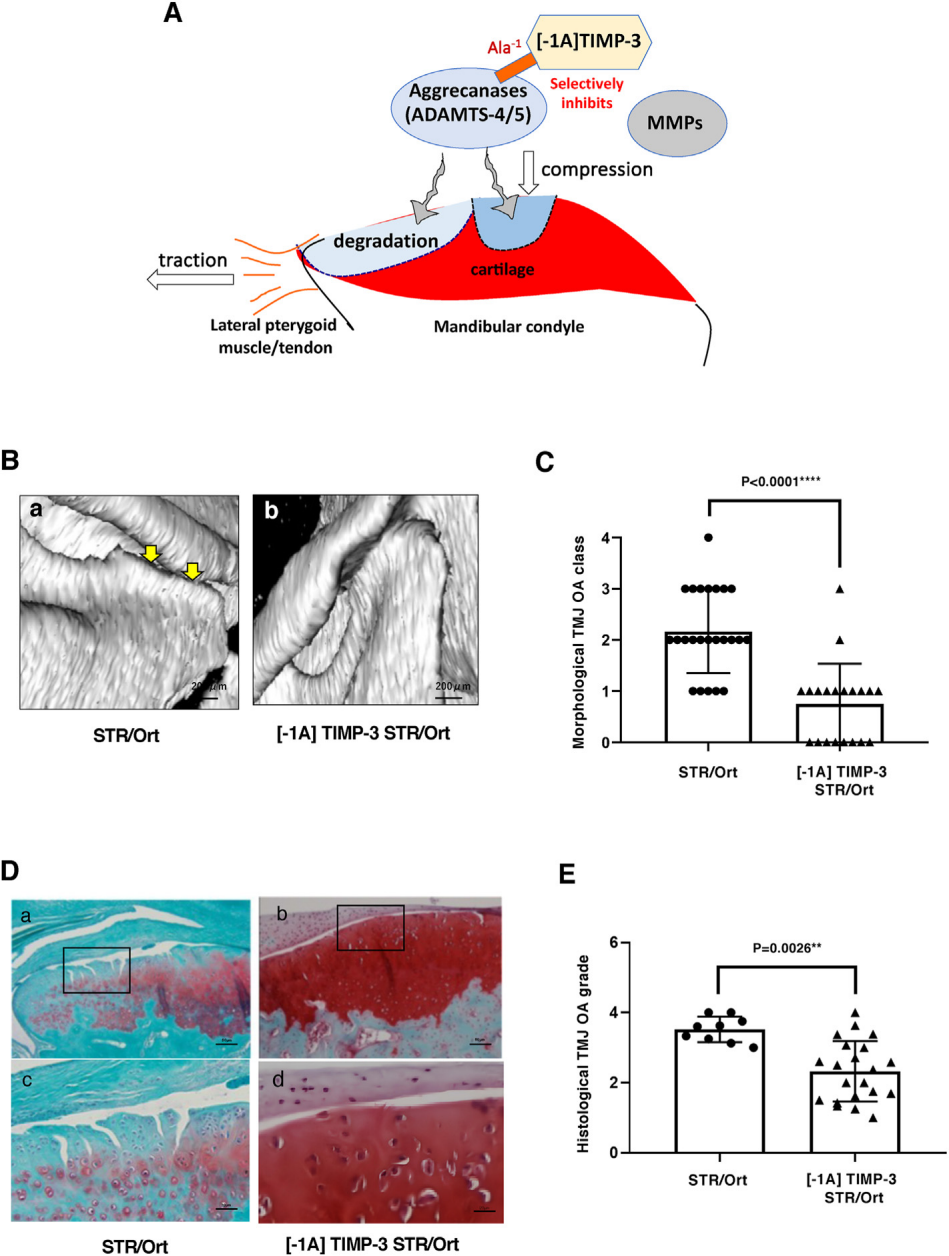
**Overexpression of [-1A] TIMP-3 reduces TMJ bone resorption and cartilage destruction in STR/ort mice.** (A) Schematic TMJ-OA protection by [-1A] TIMP-3 (B) TMJ-OA in untreated STR/ort (a), normal condylar head in [-1A] TIMP-3 STR/ort (b) (C) Morphological TMJ OA class was significant lower than in untreated STR/ort (D) Cartilage destruction was protected in [-1A] TIMP-3(a,c) compared with untreated STR/Ort mice (b,d) (a/b: bar is 200 ​μm, c/d: bar is 50 ​μm) ​(E) Histological grade of mandibular condylar head shows [-1A] TIMP-3 mice was 2.4(1–4) significantly lower than untreated STR/Ort mice of 3.5(3–4).

Having established natural occurrence of TMJ-OA in STR/Ort mice, we next investigated whether overexpressing [-1A] TIMP-3 engenders similar protection against spontaneous OA degeneration in the TMJ as previously demonstrated in STR/Ort mouse knee joints [25]. We hypothesized that selective inhibition of ADAMTS-4/5aggrecanases using [-1A] TIMP-3 [26] will protect mandibular condyle cartilage from OA degradation (Fig. 4A). 3D micro CT images of TMJ in 40-week-old untreated STR/Ort and [-1A] TIMP-3 transgenic mice(n ​= ​19) were analyzed for joint deterioration. This showed that mandibular condylar heads in [-1A] TIMP-3 transgenic STR/Ort mice appeared almost normal, whereas untreated STR/Ort mice exhibited significantly greater (p ​< ​0.0001) TMJ degeneration (Fig. 4B). Histological grading confirmed this protection, showing significantly lower OA severity in [-1A] TIMP-3 transgenic compared to untreated STR/Ort mice (p ​< ​0.0001; Fig. 4B/C). OA grades in untreated STR/Ort mice were >3 in all TMJ, while grades <3 in most joints in [-1A] TIMP-3 STR/Ort mice (p ​= ​0.0026; Fig. 4 D/E). This indicates that [-1A] TIMP-3 overexpression protects the TMJ against vertical cleft erosion and subchondral bone changes that progresses spontaneously in OA-prone STR/Ort mice (Fig. 4D/E).

### 3.3 Age-dependent TMJ cartilage destruction but not bone resorption in CBA mice

**Fig. 5:**
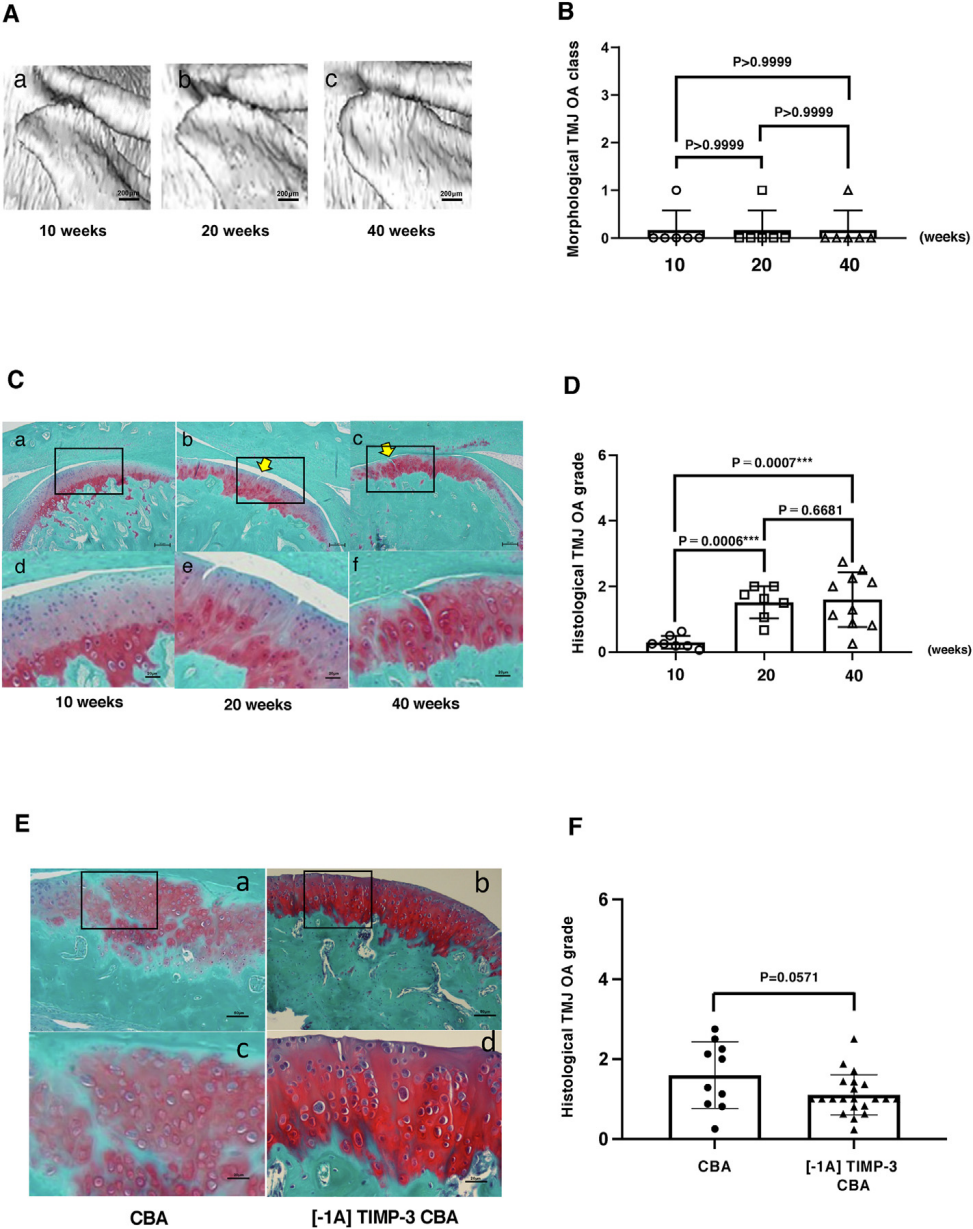
**CBA mice develop age-dependent TMJ cartilage destruction but not bone resorption and Overexpression of [-1A] TIMP-3 reduces some TMJ bone cartilage destruction in CBA mice.** (A) There were no deformities of the mandibular condyle as CBA mice progressed from 10 to 40 weeks of age. (a–c: normal, class 0). (B) Morphological score of mandibular condylar head were not observed statistically significant differences. (C) The histological changes were observed from grade 0 ​at 10 weeks, grade 1 ​at 20 weeks and grade 2 ​at 40 weeks (a–c: bar is 200 ​μm, d–f: bar is 50 ​μm). (D) Histological score of mandibular condylar head was increased between 20 and 40 weeks of age rather than 10 weeks. (E) Histological images of mandibular condylar head shows [-1A] TIMP-3 CBA of cartilage is more protective rather than untreated CBA. (a/b: bar is 200 ​μm, c/d: bar is 50 ​μm). (F) Histological evaluation of mandibular condylar head showed [-1A] TIMP-3 CBA of TMJ-OA grade reduced rather than that of untreated CBA, however p ​= ​0.0571.

μCT analysis revealed no significant morphological deformities in mandibular condyle of CBA mice at 10 (n ​= ​6), 20 (n ​= ​7) or 40 weeks (n ​= ​10) of age; all joints were classified as Class 0 or 1 (Fig. 5A/B). Histological grading, however, showed a different trend with Grade 0 TMJ-OA in 10-week-old CBA mice, which was increased significantly (only to Grade 1) at 20 weeks (p ​= ​0.0006), without further progression in 40 week-old CBA mice (n ​= ​0.668; Fig. 5C/D). Comparing untreated CBA with [-1A] TIMP-3 CBA mice showed that [-1A] TIMP-3 provided some, albeit not statically significant, protection against TMJ-OA (p ​= ​0.0571; Fig. 5E/F). μCT-based morphological classification showed no significant differences in TMJ degeneration between untreated CBA and [-1A] TIMP-3 CBA mice (Supplement. 1). Significant differences in CT morphological and histological assessments of TMJ-OA were observed between CBA and STR/Ort mice across different age (p ​< ​0.0001).

## 4 Discussion

Our studies demonstrate that the STR/Ort mouse provides a new model in which genetic susceptibility predisposes to TMJ-OA in the absence of any traumatic loading. They also show TMJ cartilage of the mandibular condylar in these mice is protected against such OA-related progressive deterioration by genetic manipulation, via targeted [-1A] TIMP-3 [26] overexpression, that engenders lower susceptibility to the actions of aggrecanases. They extend studies showing that similar inhibition protects the articular cartilage of the knee from OA changes [24,25] and emphasise that this is achieved despite the TMJ expressing a distinct structural composition to reaffirm the future utility of this mouse strain in tracking the efficacy of any new therapeutic candidates for TMJ-OA.

Our studies using a morphological scoring system, based on a human TMJ-OA classification tool, using μCT in mice, combined with histological grading served to confirm the progressive severity of OA in TMJ of STR/Ort mice as they age. These findings establish age-dependent TMJ degeneration in STR/Ort mice, implying that the genetic susceptibility to knee joint OA development also extends to the TMJ. We propose that the STR/Ort TMJ-OA model will have value in identifying new therapeutic candidates. Indeed, very little is known about the local enzymes that cause TMJ-OA destruction, especially since it differs from other joints in comprising a fibrocartilage rather than a hyaline cartilage layer [9]. Our data imply a prominent role for aggrecanases in tissue destruction in TMJ-OA, suggesting that these joint pathological processes have molecular aetiology that resembles mouse and human OA in other joints and that emerging therapies may therefore have cross-joint applicability.

The TMJ combines both hinge actions and sliding motions, with articular cartilage covered surfaces separated by a small shock-absorbing disk, which normally keeps the movement smooth. In humans, pain is thought to emerge either when the disk erodes or is moved out of proper alignment by trauma, or when the cartilage is damaged by OA. Symptomatic therapy including surgery is currently the only effective remedy, one of our aims was to explore the utility of the STR/Ort strain as a model of TMJ-OA in which μCT-based classification of degeneration and histological evaluation of joint OA can be used to objectively grade severity of disease to allow for the testing of possible treatment that prevent such TMJ joint deterioration. Our data confirm that STR/Ort mice are a useful animal model for assessing age-dependent TMJ-OA.

Our study provides clear evidence of mandibular condyle degeneration and osteophyte formation from 20 weeks of age which worsens by 40 weeks in STR/Ort mice. Using a visual system previously applied in humans, we classified morphological deterioration in mouse TMJ for the first time. This classification showed that STR/Ort mouse TMJ deterioration parallels human TMJ-OA, distinguishing normal articulations from pathological changes via μCT. Histological analysis confirmed TMJ-OA severity, correlating with μCT classifications, and demonstrated progressive proteoglycan loss and cartilage destruction in the mandibular condyle by 40 weeks in STR/Ort mice. Conversely, although there was a trend of mandibular condylar surface degradation, CBA mice showed no morphological changes with aging. Histopathological differences in OA severity between STR/Ort and CBA mice suggests that significant morphological changes appear only when histological damage reaches grade 3, which involves vertical clefts/erosion extending to the articular surface. The predictability of this progression establishes the STR/Ort strain as a valuable model for studying TMJ-OA development.

Our study also shows that STR/Ort mice do not necessarily exhibit left: right symmetry in TMJ-OA arising spontaneously, but that they do show statistically significant sex differences in OA grade. This indicates that female STR/Ort mice demonstrate greater vulnerability than males to TMJ-OA, suggests that this sexually dimorphic behaviour differs from that shown for knee OA which has been reported to develop with greater penetrance and severity in males. These findings align TMJ-OA is STR/Ort mouse more closely with human disease [30].

Standardized use of animal models is crucial for understanding disease mechanisms and validating therapeutics [31,32]. However, the scarcity of appropriate models and limited knowledge of disease mechanisms means that TMJ-OA continues to negatively impact life quality and impose financial burdens. Current models involve surgery [21,33], chemical injections [34], occlusion induction [35,36] and gene knockouts [37]; Yuan et al. [21] analyzed 160 articles (1966–2021) and found that TMJ-OA models primarily use mice, rats, rabbits, and sheep, with interventions like surgery and chemical injections, while few studies used naturally occurring TMJ-OA models. Such interventions can complicate interpretation, highlighting an advantage of spontaneous models like STR/Ort mice.

Human TMJ-OA typically begins with early articular cartilage erosion, advancing to osteophytosis, joint deformity, and finally PCR, where the mandibular condyle is almost entirely resorbed as seen in X-ray/CT images. Morphological evaluation of mandibular condyles is crucial for diagnosing advanced TMJ-OA in clinical settings but does not assess early articular cartilage clefts, which require invasive tools in animal studies. Routine use of morphological classification for mandibular condyle changes has been limited. We propose a new morphological classification system for use in STR/Ort mouse TMJ, demonstrating its applicability to clinical evaluation of human OA. Additionally, we developed TMJ-OA histological grading based on the OARSI system by Glasson et al., tailored to the TMJ's unique fibrocartilage surface layer. Our studies show that the μCT- and histology-based systems align closely and correlate well, thus reinforcing their validity.

The STR/Ort mouse develops a well-recognized natural OA in the knee that closely resembles human disease^,^ [38,39]. Recent understanding suggests that broad generalizations about OA pathogenesis may hinder the search for effective treatments. Treatment options for TMJ-OA are currently very limited to drastic surgery and there are currently no drug interventions that can slow disease progression, but intriguingly, low-intensity pulsed ultrasound (LIPUS) has been used as a non-invasive treatment that can help with TMJ disorders in both animal and human [40,41], making animal models like the STR/Ort valuable for studying primary OA. STR/Ort mice exhibit higher bone mass, especially in non-OA-prone females, while pre-OA changes in bone shape are limited to males, where knee OA develops spontaneously. This model provides crucial insights into OA mechanisms and potential treatments. Having established that STR/Ort mice develop TMJ-OA – quantifiable by morphological μCT-based classification and histological grading – we next focused on the underlying mechanisms. Previous studies suggest that anterior disc displacement leads to terminal cartilage destruction in the TMJ, with early disc repositioning alleviating degeneration in surgical models [42]. Anterior disc displacement without reduction and TMJ-OA are related to dentofacial deformities such as retro-mandible, mandibular asymmetry and anterior open bite were reported. Therefore, elucidation of TMJ-OA aetiology also has potential to prevent mandibular malformation [43,44]. Secreted molecules, particularly proinflammatory cytokines like IL-1β and TNF, are key mediators of OA pathogenesis and important targets for controlling degradation. Other cytokines, including IL-6, -8, -15, −17, and −18, are also implicated [45]. Specifically, IL-1β, IL-6, and TNF-α have been identified as crucial mediators in TMJ-OA [46,47].

Twenty-three distinct human MMPs are known, with MMP-1 and MMP-13 being the most clearly linked to OA. MMP-1 is primarily derived from the synovial joint lining, while MMP-13 is produced by cartilage chondrocytes and can degrade both collagen and proteoglycan/aggrecan. Elevated expression of MMP-2, -3, and -9, which target non-collagenous joint matrix components, has also been observed in OA [48]. Studies in TMJ have shown high levels of MMP-2, -3, -7, -9 [48,49], and −13 [50] in synovial tissue and discs of TMJ-OA patients. Bone resorption at the articular surface, particularly at the lateral pole was statistically significant at initial diagnosis of TMJ OA. Synovial fluid levels of ANG, GDF15, TIMP-1, CXCL16, MMP-3 and MMP-7 were correlated with bone apposition [51]. These findings highlight significant overlaps but also differences in cytokine and protease expression profiles, indicating that OA mechanisms in the knee/hip and TMJ may not be identical.

We have focused on whether the ADAMTS-4/5 aggrecanases contribute to degrading aggrecan as a component of TMJ-OA. We have previously found that STR/Ort mice overexpressing [-1A] TIMP-3 exhibit protection against knee OA development. μCT data showed that untreated STR/Ort controls had severe subchondral sclerosis on the medial side of the knee joint, which was abrogated in [-1A] TIMP-3–transgenic STR/Ort mice [21]. In this study, we tested aggrecanase involvement in TMJ-OA by generating transgenic mice overexpressing [-1A] TIMP-3 on the STR/Ort genetic background. We examined TMJ-OA development using our μCT-based classification and histological grading of progression to test the hypothesis that age-related TMJ-OA in STR/Ort mice relies on the selective action of ADAMTS-4/5 aggrecanases. Our findings support the notion that ADAMTS-4/5 are involved in the spontaneous TMJ-OA that arises in STR/Ort mice and that these joints exhibit both similar pathological processes and molecular aetiology to OA evident in other joints. While [-1A] TIMP-3 may inhibit other ADAMs, these data support the use of STR/Ort mice as a valuable resource in the study of TMJ-OA, with the potential to identify new therapeutic strategies to protect against TMJ-OA.

## Author contributions

All authors participated in the study design, interpretation of results, and drafting the manuscript or critically revising it for re-levant intellectual content. Kazuhiro Ooi designed the study, performed the research work and data analysis and contributed to writing the original and final draft. Kazuhiro Yamamoto contributed to writing the original and final draft and revising it critically for intellectual content. Yutaka Kobayashi and Anders Jensen contributed to the research work and performed the data analysis. Behzad Javaheri, Ioannis Kanakis, Takao Sakai, Fadi Jarad, and Hiroyuki Nakamura contributed to writing the final draft and revising the manuscript. George Bou-Gharios, Andrew A. Pitsillides and Shuichi Kawashiri supervised and encouraged the study and contributed to data analysis, writing the final draft, and revising it critically for important intellectual content.

## Declaration of competing interest

The authors declare no competing interests.

## Appendix A Supplementary data

Multimedia component 1 (A) Morphological mandibular condylar head showed both untreated CBA and [-1A] TIMP-3 CBA were similar. (B) There was no significant difference of morphological TMJ-OA scoring between untreated CBA and [-1A] TIMP-3 CBA. Multimedia component 1
