# Supplementary figures and images for: Temporomandibular joint degeneration arises spontaneously in STR/ort mice and is prevented by targeted aggrecanase inhibition
Source: Osteoarthr Cartil Open. 2025 Mar 11;7(2):100599. doi: 10.1016/j.ocarto.2025.100599 (PMC11981737; doi:10.1016/j.ocarto.2025.100599)

# Supplement.1

A

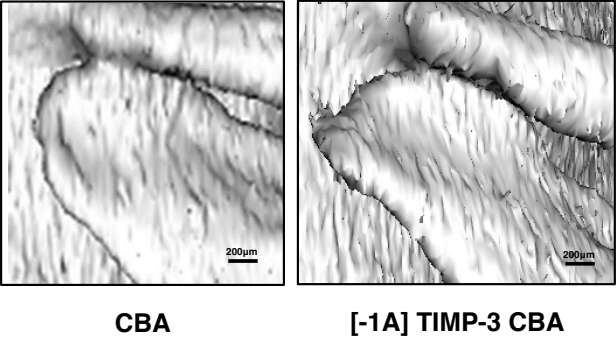

B

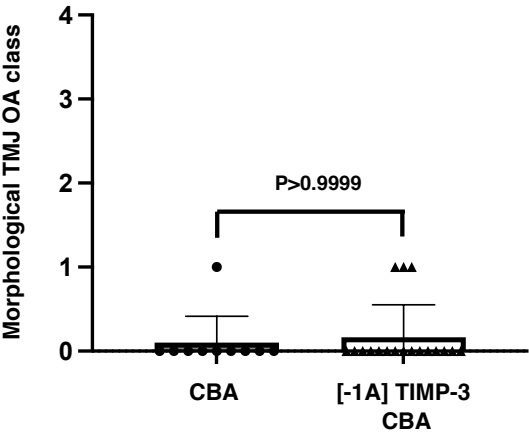

Supplement: Multimedia component 1 — (A) Morphological mandibular condylar head showed both untreated CBA and [-1A] TIMP-3 CBA were similar. (B) There was no significant difference of morphological TMJ-OA scoring between untreated CBA and [-1A] TIMP-3 CBA. [file mmc1.pdf]
